# Shikimate Metabolic Pathway Engineering in *Corynebacterium glutamicum*

**DOI:** 10.4014/jmb.2106.06009

**Published:** 2021-08-03

**Authors:** Eunhwi Park, Hye-Jin Kim, Seung-Yeul Seo, Han-Na Lee, Si-Sun Choi, Sang Joung Lee, Eung-Soo Kim

**Affiliations:** 1 Department of Biological Sciences and Bioengineering, Inha University, Incheon 22212, Republic of Korea; 2 STR Biotech Co., Ltd., Chuncheon 24232, Republic of Korea

**Keywords:** Shikimate, metabolic pathway engineering, *Corynebacterium*, genome editing, fed-batch fermentation

## Abstract

Shikimate is a key high-demand metabolite for synthesizing valuable antiviral drugs, such as the anti-influenza drug, oseltamivir (Tamiflu). Microbial-based strategies for shikimate production have been developed to overcome the unstable and expensive supply of shikimate derived from traditional plant extraction processes. In this study, a microbial cell factory using *Corynebacterium glutamicum* was designed to overproduce shikimate in a fed-batch culture system. First, the shikimate kinase gene (*aroK*) responsible for converting shikimate to the next step was disrupted to facilitate the accumulation of shikimate. Several genes encoding the shikimate bypass route, such as dehydroshikimate dehydratase (QsuB), pyruvate kinase (Pyk1), and quinate/shikimate dehydrogenase (QsuD), were disrupted sequentially. An artificial operon containing several shikimate pathway genes, including *aroE*, *aroB*, *aroF*, and *aroG* were overexpressed to maximize the glucose uptake and intermediate flux. The rationally designed shikimate-overproducing *C. glutamicum* strain grown in an optimized medium produced approximately 37.3 g/l of shikimate in 7-L fed-batch fermentation. Overall, rational cell factory design and culture process optimization for the microbial-based production of shikimate will play a key role in complementing traditional plant-derived shikimate production processes.

## Introduction

Shikimate is a key metabolic intermediate in the shikimate pathways that are indispensable for maintaining the normal metabolism in plants, animals, and microorganisms. It is also a natural substance with high industrial value as a precursor to oseltamivir, an anti-influenza drug known as Tamiflu [1, 2]. Shikimate can be used as an intermediate or versatile chiral precursor to synthesize bio-renewable aromatics and stabilize metal nanoparticles. Current methods of shikimate production include extraction from plant star anise (*Illicium verum*), chemical synthesis, and fermentation of genetically modified microorganisms [1]. Shikimate production by plant-based extraction and chemical synthesis suffers from low yield and high cost, making it difficult to meet the increasing world demand for Tamiflu since the advent of swine and avian influenza [2-4]. Therefore, the fermentation of an engineered microbial strain utilizing renewable resources, such as glucose, would be an alternative sustainable approach [2-7].

**Fig. 1:**
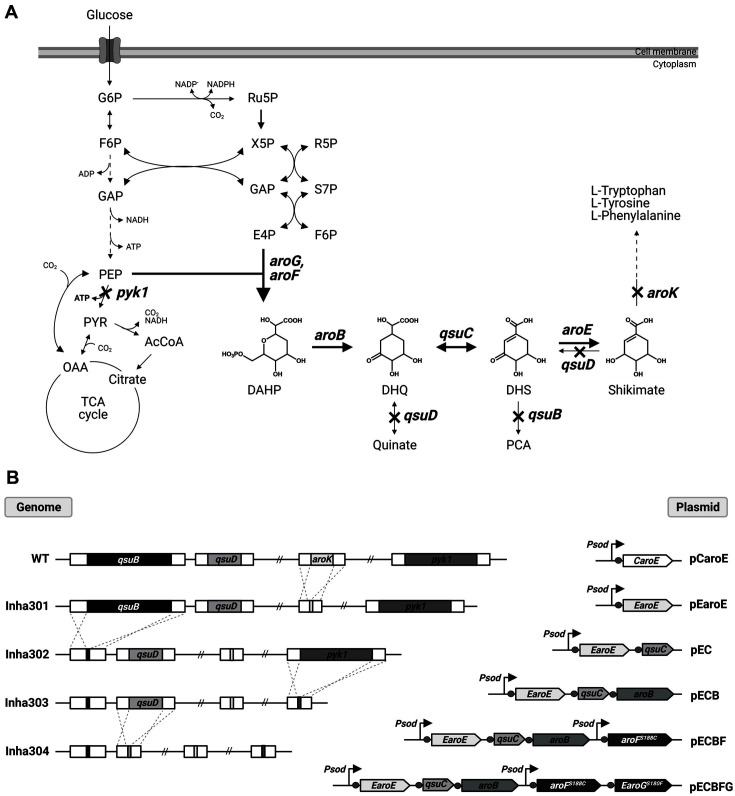
Pathway engineering strategy for shikimate production in *C. glutamicum*. (**A**) Shikimate metabolic pathway in *C. glutamicum*. The bold arrows and crosses indicate the steps for which corresponding genes were overexpressed and disrupted, individually. The dashed lines represent several catalytic steps. The genes involved in each step are shown in italics. G6P, glucose-6-phosphate; F6P, fructose-6-phosphate; GAP, glyceraldehyde-3-phosphate; PEP, phosphoenolpyruvate; PYR, pyruvate; AcCoA, acetyl-CoA; OAA, oxaloacetate; Ru5P, ribulose-5-phosphate; X5P, xylulose-5-phosphate; R5P, ribose- 5-phosphate; S7P, sedoheptulose-7-phosphate; E4P, erythrose-4-phosphate; DHAP, 3-deoxy-D-arabinoheptulosanate-7- phosphate; DHQ, 3-dehydroquinate; DHS, 3-dehydroshikimate; PCA, protocatechuate. Genes and corresponding enzymes are as follows: *pyk1*, pyruvate kinase 1; *aroF* and *aroG*, DAHP synthase; *aroB*, 3-dehydroquinate synthase; *qsuC*, dehydroquinate dehydratase; *aroE*, shikimate dehydrogenase; *aroK*, shikimate kinase; *qsuD*, quinate/shikimate dehydrogenase; *qsuB*, dehydroshikimate dehydratase. (**B**) Gene disruption in *C. glutamicum* ATCC13032 and plasmid construction. The constructed plasmids were introduced and replicated in Inha304, yielding Inha305, Inha306, Inha307, Inha308, Inha309, and Inha310, respectively. *CaroE* and *EaroE*, shikimate dehydrogenase from *C. glutamicum* ATCC13032 and *E. coli* K-12, respectively; *aroF^S188C^*, DAHP synthase carrying S188C mutation; *EaroG^S180F^*, DAHP synthase carrying S180F mutation.

Shikimate is typically synthesized by a series of enzyme-led stepwise bioconversions shown in Fig. 1. 3-Deoxy-D-arabino-heptulosonate-7-phosphate (DAHP) is first produced by the condensation of phosphoenolpyruvate (PEP) and erythrose-4-phosphate (E4P), followed by sequential conversions to 3-dehydroquinate (DHQ), 3-dehydroshikimate (DHS), and shikimate. Shikimate is then transformed further to shikimate-3-phosphate by shikimate kinase encoded by *aroK*. Shikimate-3-phosphate is then transformed into chorismate, which is finally converted to phenylalanine, tyrosine, tryptophan, and other aromatic products. Several metabolic pathway-engineering attempts have been applied to produce useful aromatic compounds and other chemicals derived from the shikimate pathway [8, 9]. Examples include salicylic acid (SA) [10], muconic acid [11-14], and 3-dehydroshikimate (DHS) [15], which are derived from chorismate and used as precursors in aspirin synthesis.

*Corynebacterium glutamicum* is a general-regarded-as-safe (GRAS) microorganism that is used industrially for amino acid production. The microorganism has been reported to produce more than two million tones of L-glutamate and one and a half tons of L-lysine annually [16-18]. It has also been used as a host to produce L-arginine [19], L-cysteine [20], anthocyanin [21], hydroxybenzoic acid [22], taurine [23], and 3-hydroxypropionic acid (3-HP)[24], as well as the lignocellulose-based production of fuels and chemicals in biorefineries [25]. Moreover, rationally genome-redesigned *C. glutamicum* produced approximately 38 g/L of muconic acid in 7-L fed-batch fermentation, suggesting that *C. glutamicum* could be an ideal bacterial host for an artificial cell factory design [12].

In the present study, a shikimate high-producing *C. glutamicum* strain was constructed by shikimate pathway engineering for 7-L fed-batch fermentation. These results suggest that the *Corynebacterium* cell factory design for a shikimate-overproducing strain would be valuable for constructing a microorganism-based high-producing strain for aromatic compounds with industrial value.

## Materials and Methods

### Bacterial Strains and Culture Conditions

Table 1 lists all bacterial strains used in this study. *E. coli* DH5α was used for genetic manipulation and grown in Luria-Bertani (LB) medium at 37°C with the appropriate antibiotics. The *C. glutamicum* strains were cultivated in a brain heart infusion (BHI) medium and BHIS (BHI medium containing 91 g/l sorbitol) at 30°C. The preparation of shikimate production media and cultivations for *C. glutamicum* were performed as previously described [12].

### Construction of Plasmid and Strains

Table 1 presents the constructed plasmids, and Table S1 lists all primer pairs used in this study. For markerless target gene disruption, pK19mobsacB was used, and the plasmids of pCaroE, pEaroE, pE, pEC, pECB, pECBF, and pECBFG were constructed for gene overexpression, which was controlled under the sod promoter. Target gene disruption was verified by colony PCR using each primer set. For overexpression, the *aroE* genes from *C. glutamicum* and *E. coli* were amplified by PCR and inserted into pSK003 using an In-Fusion cloning kit (TaKaRa, Japan), yielding pCaroE and pEaroE, respectively. The *qsuC*, *aroB* genes were cloned individually into pEaroE to generate pEC and pECB. A three-way PCR method was used for the site-directed mutagenesis of the *aroF* gene from *C. glutamicum* and *aroG* gene from *E. coli*, and the aroF^S188C^ fragment and EaroG^S180F^ were inserted sequentially into pECB, yielding pECBF and pECBFG. The constructed plasmids were introduced individually into *C. glutamicum* strains via electroporation as described [12].

### Shikimate and Dehydroshikimate (DHS) Analyses

Cultured broth samples were centrifuged (4°C, 15,000 RPM for 7 min), and only the supernatant was diluted and purified using a membrane filter (Nylaflo nylon membrane filter) for high-performance liquid chromatography (HPLC). The concentrations of shikimate and DHS were determined by HPLC using an Aminex HPX-87H column (Bio-Rad). The column was heated to 50°C to detect shikimate and DHS. The mobile phase was 2.5 mM H_2_SO_4_, and the flow rate was 0.5 ml/min for shikimate. Shikimate and DHS were detected at 215 nm and 236 nm, respectively.

## Results and Discussion

### Engineering of the Shikimate Pathway in *C. glutamicum* ATCC13032

**Fig. 2:**
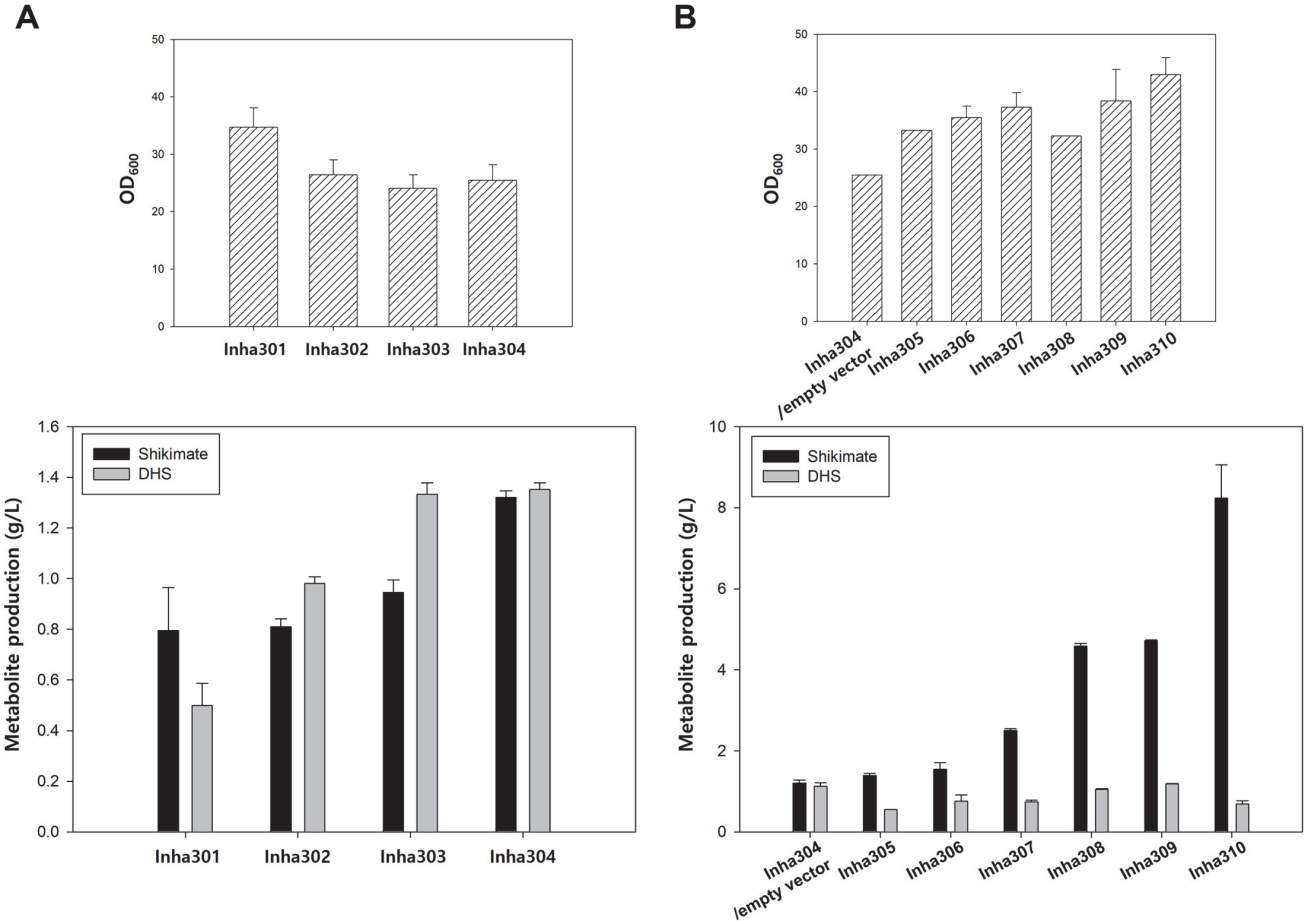
Metabolite production yield in recombinant *C. glutamicum* strains. (**A**) Cell growth (top) and shikimate and dehydroshikimate (DHS) production yield (bottom) in target gene deleted *C. glutamicum* strains; Inha301, Inha302, Inha303, and Inha304. (**B**) Cell growth (top) and HPLC analysis of shikimate and dehydroshikimate (DHS) production (bottom) in the Inha304 harboring empty vector, pSK003, and target gene overexpressed, individually; Inha305, Inha306, Inha307, Inha308, Inha309, and Inha310. The values represent the means and standard deviations of duplicate cultivations.

The shikimate pathway of *C. glutamicum* was engineered to build a shikimate high-production strain. To construct a *Corynebacterium* strain that could accumulate shikimate, *aroK* (NCgl1560) encoding shikimate kinase, which mediates the conversion of shikimate to shikimate-3-phosphate, was first disrupted (named Inha301) and confirmed to produce 0.8 g/l of shikimate (Figs. 1 and 2A). *qsuB* (NCgl0407) was then deleted (named Inha302) to accumulate the key precursor, dehydroshikimate dehydrate (DHS), blocking DHS conversion to protocatechuate (PCA). Approximately, 0.98 g/l of DHS was accumulated in the Inha302 strain, which is 1.97 times higher than 0.5 g/l of DHS produced in Inha301 (Fig. 2A). In addition, the pyruvate kinase gene (Pyk1, NCgl2008) was removed (named Inha303) for the build-up of phosphoenolpyruvate (PEP), resulting in 1.33 g/l of DHS and 0.95 g/l of shikimate (Fig. 2A). Finally, the quinate/shikimate dehydrogenase gene (*qsuD*) involved in the conversion of 3-dehydroquinate (DHQ) and quinate was deleted (named Inha304) to produce 1.32 g/l of shikimate (Fig. 2A). Thus, the sequential elimination of the *aroK*, *qsuB*, *pyk1*, and *qusD* genes could be inferred by a reasonable pathway engineering method for shikimate high production.

### Overexpression of the Shikimate Pathway Genes to Enhance Shikimate Production

Several key genes involved in shikimate biosynthesis, such as *aroE*, *qsuC*, *aroB*, *aroF*, and *aroG*, were overexpressed sequentially in the above-constructed four-genes KO stain, Inha304, to maximize the metabolite flux in the shikimate pathway. Five shikimate pathway genes (*aoE*, *qsuC*, *aroB*, *aroF*, and *aroG*) were over-expressed as a single operon under the strong sod promoter, resulting in the simple and rapid construction of a shikimate high-production strain.

Because significant amounts of DHS accumulated in Inha304, as shown in Fig. 2A, the over-expression of AroE, a shikimate dehydrogenase involved in bioconversion from DHS to shikimate, was first attempted in Inha304. For more efficient AroE expression, *C. glutamicum*-derived *aroE* (NCgl1567) and *E. coli*-derived *aroE* were introduced in Inha304 to build Inha305 and Inha306, respectively, and compare the conversion rate to shikimate. As shown in Fig. 2B, 1.55 g/l of shikimate was detected in Inha306, which is 1.11 times higher than 1.39 g/l of shikimate in Inha305, suggesting that AroE of *E. coli* might have better enzyme kinetic characteristics. The dehydroquinate dehydrate gene (*qsuC*, NCgl0408) involved in the reaction between 3-dehydroquinate (DHQ) and DHS, and the dehydroquinate synthase gene (*aroB*, NCgl1559) involved in bioconversion from 3-deoxy-D-arabinoheptulosanate to DHQ were sequentially expressed in Inha306, resulting in Inha307 and Inha308, respectively. Inha307 showed a 1.6-fold increase in shikimate production (2.51 g/l) compared to Inha306 (1.55 g/l), and Inha308 showed a 1.4- and 1.8-fold increase in DHS (1.05 g/l) and shikimate (4.59 g/l) production compared to Inha307, respectively (Fig. 2). Point mutations to the DHAP synthases *aroF* and *aroG*, which are involved in the biosynthesis of DHAP from PEP and E4P, have been reported to induce a higher resistance to feedback inhibition [26, 27]. Hence, the Inha309 strain containing *C. glutamicum*-derived *aroF* with a serine to cysteine mutation at the 188 position, and the Inha310 strain containing the *E. coli*-derived *aroG* with a serine to phenylalanine mutation at the 180 position, were used. As a result, Inha309 produced 1.19 g/l of DHS and 4.72 g/l of shikimate, and Inha310 exhibited the highest production yields of 0.69 g/l of DHS and 8.23 g/l of shikimate (Fig. 2B). These results suggest that the pathway engineering strategy described here is a fast and effective approach for shikimate over-production in *C. glutamicum*.

### Fed-Batch Fermentation of Inha310

**Fig. 3:**
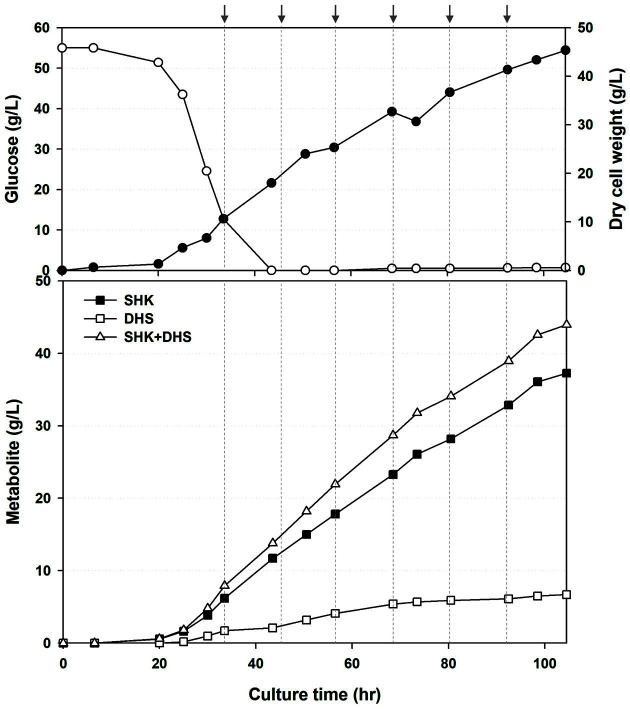
Time-course profiles of cell growth (Dry cell weight) and glucose concentrations and metabolite production by Inha310 in a 7-L bioreactor. The feeding medium was injected at 33.5, 44.5, 56.5, 68.5, 80.5, and 92.5 h (indicated with gray arrow), at a rate of 0.189 ,0.246, 0.321, 0.416, 0.548, and 0.699 ml/min, respectively.

A 5 L batch fermentation was performed to calculate the feeding medium flow rate for the fed-batch fermentation of the Inha310 strain. The formula for calculating the feed medium flow rate is as follows. [*F*] = *Qs*[*V*]/[*S*0] [28]. Qs, V, and S0 are the initial glucose concentration (g/l)/glucose consumption rate (h), the initial volume of the feeding medium, and glucose concentration of the feeding medium, respectively. Therefore, Inha310 consumed all the glucose in a 2 L feeding medium containing 55 g/l of glucose for 24 h. Hence, the calculated feeding medium flow rate was at least 0.185 ml/min based on the above formula. After 16-h of 1^st^ seed culture and 6-h of 2^nd^ seed culture, the Inha310 culture was inoculated into a 5 L fermenter. The feeding medium was added at 33.5 h at a rate of 0.189 ml/min whenever the glucose concentration decreased below 10 g/l during 104.5 h fermentation. As a result, the Inha310 continued to grow during the entire fed-batch fermentation period, and the production of shikimate increased accordingly, finally reaching up to 37.3 g/l (Fig. 3).

Similar strategies for shikimate overproduction have been attempted in *E. coli* and *Bacillus subtilis* strains, but at a low titer to meet commercial applications [29]. Among the results reported thus far, one of the best titers related to shikimate production is through metabolically engineered *C. glutamicum* in a growth-arrested cell reaction with 141 g/l of shikimate [2]. The shikimate titer should not be compared directly because the fed-batch system described here is quite different from the previously reported cell-arrest culture process. In the future, improvement in the key enzymes through protein engineering, optimization of transcription and translation processes through promoter and ribosomal binding site (RBS) optimization, and stable chromosomal DNA integration will enable the establishment of a more stable shikimate hyper-production strain.

## Supplemental Materials

Supplementary data for this paper are available on-line only at http://jmb.or.kr.

## Figures and Tables

**Table 1:** Strains and plasmids used in this study.

Strain or plasmid	Characteristics	Sources or reference
*C. glutamicum* ATCC13032		
Inha301	△*aroK*(NCgl1560)	This study
Inha302	△*aroK*(NCgl1560)△*qsuB*(NCgl0407)	This study
Inha303	△*aroK*(NCgl1560)△*qsuB*(NCgl0407)△*pyk1*(NCgl2008)	This study
Inha304	△*aroK*(NCgl1560)△*qsuB*(NCgl0407)△*pyk1*(NCgl2008)△*qsuD*(NCgl0409)	This study
Inha305	Inha304 with pCaroE	This study
Inha306	Inha304 with pEaroE	This study
Inha307	Inha304 with pEC	This study
Inha308	Inha304 with pECB	This study
Inha309	Inha304 with pECBF	This study
Inha310	Inha304 with pECBFG	This study
Plasmid		
pK19mobsacB	Vector for the construction of disruption mutants of *C. glutamicum*	[30]
pSK003	*E. coli*-*C. glutamicum* shuttle vector harboring *sod* promoter)	This study
pCaroE	pSK003 carrying the *aroE* gene from *C. glutamicum* ATCC13032	This study
pEaroE	pSK003 carrying the *aroE* gene from *E. coli* K-12	This study
pEC	pEaroE carrying the *qsuC* gene (NCgl0408) from *C. glutamicum* ATCC13032	This study
pECB	pEC carrying the *aroB* gene (NCgl1559) from *C. glutamicum* ATCC13032	This study
pECBF	pECB carrying the *aroF* gene (NCgl0950) carrying S188C mutation	This study
pECBFG	pECBF carrying the *aroG* gene from *E. coli* K-12 carrying S180F mutation	This study
