# Supplementary material for: Shikimate Metabolic Pathway Engineering in Corynebacterium glutamicum
Source: J Microbiol Biotechnol. 2021 Aug 3;31(9):1305–10. doi: 10.4014/jmb.2106.06009 (PMC9705862; doi:10.4014/jmb.2106.06009)
Supplement: Supplementary file 1 [file jmb-31-9-1305-supple.pdf]

J. Microbiol. Biotechnol.
https://doi.org/10.4014/jmb.2106.06009

Table S1. Primer pairs for the construction of plasmids and target gene amplification used in this study

Purpose	Forward(F)<br>Reverse(R)
aroK disruption upstream	TGATTACGCCAAGCTCATGACCAACGGTGAAACCC<br>CAGGAGCATTCAGAGGTGTC
aroK disruption downstream	CTCTGAATGCTCCTGGGCAGCAGTGTTGCATCATC<br>GCAGGCATGCAAGCTCTTCCAAGGACTTGCCGTTT
aroK recombination check	TCCTGGATGACAACGGCGTA<br>CAATTCATCCCAGCACTGCC
	GAGCAACATTGACACCTACC<br>GTTAATCAGGTGCCCGGTTT
qsuB disruption upstream	TGATTACGCCAAGCTGGTTTCGTCCCAGTTATCGC<br>GCAGGCATGCAAGCTCTCTTCTTCCACACCTTCGA
qsuB disruption downstream	GCAGGTCGACTCTAGGAGAACGGCGAATTCCTCCA<br>CCGGGGATCCTCTAGGGTTAATCACGCAACCGATG
qsuB recombination check	TCCAGTGCGATTTCCGTTTC<br>CCTCAACGGTAGGAAGCTCA
	AGCTGCAGATGCTGGAT<br>TCAGGTTTGGGCCGTTGA
qsuD disruption upstream	TGATTACGCCAAGCTAACCTGAACATGCTGGGCAA<br>GAACAGTCGGAAGGCTAGGCCGAGGAGAATACTGT
qsuD disruption downstream	GCCTTCCGACTGTTCACC<br>GCAGGCATGCAAGCTCTTCATTAGGCGATCACCGG
qsuD recombination check	TTGATCCCCGGGATCCCCCAACAACAATTAGAAAT<br>CGCATGCGGGAGACGT
	CCGTTTCGCGGTCGATA<br>CTGGTGAGCCAAAGAGGC
pyk1 disruption upstream	TGCCTGCAGGTCGACTAGAGCTTTGCAATCCTTGT<br>CGGGGATCCTCTAGATGGTTCAGAAGCGCGCAATC
pyk1 disruption downstream	CCCGGGTACCGAGCTGAATTCCATCTGCACTAGCC<br>CGGCCAGTGAATTCGCGGTACAAAAAGCTTCCTCT
pyk1 recombination check	TTGGGTATCGAAGAGGAAAGCGGTG<br>TACTTGGGTCCCATTCCGATTAGGG
	TTGTACTCAGGCATTGCTAAAAGAG<br>CACCATCGTCTCCGCAGTAGTGTTT
CaroE amplification	GATTTTTTACCCATATTGGGTTCTCACATCACTCA<br>ATTAGGAATTCCATATTAGTGTTCTTCTGAGATGC
EaroE amplification	GATTTTTTACCCATAATGGAAACCTATGCTGTTTT<br>ATTAGGAATTCCATATCACGCGGACAATTCCTCCT
qsuC amplification	TTGATCCCCGGGATCCCCCAACAACAATTAGAAAT<br>AGAGCTATCGGGATCCTACTTTTTGAGATTTGCCA
aroB amplification	TCCCGATAGCTCTAGTTACAAGCGAAAGGCAACGT<br>CGCGTCATGCTCTAGTTAGTGGCTGATTGCCTCAT
aroF amplification	AGGAGACACAACATATGAGTTCTCCAGTCTCACT<br>ATTAGGAATTCCATATTACTTGGCTGCTGCTCGGC
aroF mutation	ATGCCAATTGGTTTCAAGAA<br>TTGAAACCAATTGGCATGCACATCCCAGAAGCCAG

aroG amplification	TTGATCCCCGGGATCTAAGATAAGTATGGCAACAC<br>AGAGCTATCGGGATCTTACCCGCGACGCGCTTTTA
aroG mutation	CTTTTCTGTCCGGTCGGCTTC<br>CCGGACAGAAAAGCCCTGAT
